# Diagnostic value of ultrasound features and sex of fetuses in female patients with papillary thyroid microcarcinoma

**DOI:** 10.1038/s41598-018-26003-5

**Published:** 2018-05-14

**Authors:** Chun-jie Hou, Ran Wei, Jing-lan Tang, Qiao-hong Hu, Hong-feng He, Xiao-ming Fan

**Affiliations:** 1 Department of Ultrasound, Zhejiang Provincial People’s Hospital, Hangzhou, China; 2 People’s Hospital of Hangzhou Medical College, Hangzhou, China; 3 Department of General Surgery, Huashan Hospital & Cancer Metastasis Institute, Fudan University, Shanghai, China

## Abstract

Little work has been done on the prediction of papillary thyroid microcarcinoma in female patients who have given birth to children, which may be different from other people. We performed a retrospective review of female patients who underwent thyroidectomy, aiming at identifying special predictors of papillary thyroid microcarcinoma in female patients who have given birth to children. Univariate analysis was used to identify potential covariates for the prediction of papillary thyroid microcarcinoma. Multivariable logistic regression analysis was used to identify independent predictors and construct a regression model based on a training cohort (246 patients) and then the regression model was validated using an independent cohort (80 patients). We found that having not more than one boy, taller-than-wide shape, poorly defined margin, marked hypoechogenicity, and microcalcification were independent risk factors for the papillary thyroid microcarcinoma in multivariate analyses. The combined predictive formula had a high predictive effect for papillary thyroid microcarcinoma (AUC = 0.938 for training cohort and 0.929 for validation cohort, respectively). The combined predictive formula has clinical value in the prognosis of papillary thyroid microcarcinoma and it may be simple and effective to ask fertility condition of patients to increase the US diagnosis accuracy of papillary thyroid microcarcinoma.

## Introduction

Thyroid cancer is the most common endocrine malignancy, and papillary thyroid microcarcinoma (PTMC) that measure 1.0 cm or less is the most common type of thyroid cancer. Although an active surveillance approach was suggested by American Thyroid Association (ATA) guidelines as a preferred option for parts of patients with PTMC, who had no evidence of invasion, metastases, or cytological or molecular aggressive features^1^, the malignancy rates of thyroid nodules with diameters less than 1 cm do not decrease with decreasing size^2^. Instead, some studies showed subcentimeter thyroid nodules were associated with higher risk of malignancy including an aggressive pathologic course compared with larger thyroid nodules^3^.

Fine-needle aspiration (FNA) is the standard approach for differentiating benign nodules from malignant ones. Nevertheless, FNA has limitations in subcentimeter nodules as a result of its high rates of inadequate cytological specimens and false positive findings, especially for nodules less than 5 mm^4^. Therefore, it is essential to find new methods to improve the accuracy of PTMC diagnosis. Large numbers of studies have been published which have discussed conventional ultrasound (US) features for assessing thyroid nodules as malignant or benign. According to those studies, some useful sonographic features for the differential diagnosis have been proposed, including a taller-than-wide shape, a poorly defined boundary, microcalcification, and marked hypoechogenicity^5^. However, no conventional sonographic feature has both a high positive predictive value (PPV) and high sensitivity for thyroid cancer. A recent report has shown that PTMC was found most often in female patients aged >30 years old, most of whom have given birth to children^6^. Nevertheless, little work has been done on the prediction of PTMC in female patients who have given birth to children, which may be different from other people.

Leptin is a neuroendocrine hormone which is produced by white adipose tissue cells^7^. It was shown that circulating leptin level was inversely associated with the risk of thyroid cancer^8^. Leptin could act through JAK/STAT3, PI3K/AKT and ERK/MAPK pathways, resulting in the tumorigenesis of thyroid cancer^9,10^. Recent studies also found that females who delivered baby girls had higher levels of leptin than women with male fetuses during pregnancy and after delivery^11^. Therefore, the gender of fetus may serves as a predictor for thyroid cancer. The combination of US features and the gender of fetus may have higher predictive effect. Therefore, the purpose of this study was to find the special risk factors for PTMC in female patients who have given birth to children, and analyze their predictive effects.

## Results

### Baseline characteristics of patients with benign and PTMC nodules

For the training cohort, general characteristics and fertility condition of patients with benign (≤1 cm) and PTMC nodules were shown in Tables 1 and 2. 246 female patients who have children were included in the training cohort. Based on histopathology, half of them (n = 123) had benign nodules and the other (n = 123) had PTMC nodules. The mean age of the all patients was 51.46 years (range 26–80 years). Age (≥45) (83.74% vs. 65.04%, P = 0.001), diabetes mellitus (17.07% vs. 5.69%, P = 0.008) and TSH (1.64 ± 1.10 vs. 1.96 ± 1.37, P = 0.045) between two groups showed significant difference. By contrast, the levels of glucose, TC, TG, HDL, LDL and LDH were not associated with the PTMC nodules. As to fertility condition, patients who have at least two girls were more likely to have PTMC nodules (P < 0.001). Similarly, patients who have not more than one boy have higher incidence of PTMC nodules (*P* < *0.001*).

**Table 1:** Baseline patient characteristics.

Variables^a^	All patients N = 246	Benign N = 123	PTMC N = 123	P value
Age(≥45)	183(74.39%)	103(83.74%)	80(65.04%)	0.001
Body mass index(kg/m^2^)	24.73 ± 14.33	23.82 ± 3.30	25.63 ± 19.99	0.323
Hypertension	58(23.58%)	36(29.27%)	22(17.89%)	0.050
Diabetes mellitus	28(11.38%)	21(17.07%)	7(5.69%)	0.008
Time between diagnosis and surgery (months)	20.39 ± 44.68	26.67 ± 57.74	14.10 ± 24.41	0.028
Length of stay (days)	7.80 ± 2.05	7.30 ± 1.69	8.31 ± 2.24	<0.001
TSH	1.80 ± 1.25	1.64 ± 1.10	1.96 ± 1.37	0.045
Glucose	5.38 ± 1.08	5.48 ± 1.08	5.28 ± 1.08	0.146
TC	5.03 ± 0.94	5.04 ± 0.94	5.02 ± 0.95	0.832
TG	1.53 ± 1.24	1.64 ± 1.46	1.43 ± 0.98	0.198
HDL	1.35 ± 0.31	1.35 ± 0.29	1.35 ± 0.33	0.807
LDL	2.95 ± 0.73	2.95 ± 0.71	2.96 ± 0.76	0.915
LDH	174.49 ± 33.79	175.53 ± 35.99	173.45 ± 31.53	0.634

^a^Continuous data are shown as meanstandard deviation; categoric data as number (%). Data were missing for some patients TC = total cholesterol; TG = triglyceride; HDL = high density lipoprotein; LDL = low density lipoprotein; LDH = lactate dehydrogenase.

**Table 2:** Fertility of condition of patients with benign and PTMC.

Variables^a^	All patients N = 246	Benign N = 123	PTMC N = 123	P value
Have only boy(s)	105(42.68%)	56(45.53%)	49(39.84%)	0.439
Have only girl(s)	73(29.67%)	35(28.46%)	38(30.89%)	0.780
Have at least one boy	173(70.33%)	88(71.54%)	85(69.11%)	0.780
Have at least one girl	141(57.32%)	67(54.47%)	74(60.16%)	0.439
Have more than one baby	113(45.93%)	59(47.97%)	54(43.90%)	0.609
Have not more than one boy	29(11.79%)	99(80.49%)	122 (99.19%)	<0.001
Have at least two girls	40(16.26%)	5(16.26%)	24(16.26%)	<0.001
Number of babies (1:2:3:4)	132:82:31:1	63:38:21:1	69:44:10:0	0.103

^a^Categoric data as number (%).

### US characteristics

For the training cohort, we observed a total of 310 subcentimeter nodules (165 benign nodules and 145 PTMC nodules) which had histopathology evidence. The US characteristics of these nodules were not missing. There were significant differences in the shape, margins, echogenicity, microcalcification and echostructure between benign and PTMC nodules (*P* < 0.001) (Table 3).

**Table 3:** Conventional US features of benign nodules and PTMC. ^a^Categoric data as number (%).

Variables^a^	All nodules n = 310	Benign n = 165	PTMC N = 145	P value
***Shape***				<0.001
Wider-than-tall	166(53.55%)	139(84.24%)	27(18.62%)	
Taller-than-wide	144(46.45%)	26(15.76%)	118(81.38)	
***Margins***				<0.001
Well-defined	171(55.16%)	139(84.24%)	32(22.07%)	
Poorly-defined	139(44.84%)	26(15.76)	113(77.93)	
***Echogenicity***				<0.001
None	5(1.61%)	5(3.03%)	0(0.00%)	
Marked-hypoecogenicity	80(25.81%)	8(4.85%)	72(49.65%)	
hypoecogenicity	199(64.19%)	127(76.97%)	72(49.65%)	
Iso-ecogenicity or hyperecogenicity	26(8.39%)	25(15.15%)	1(0.70%)	
***Calcification***				<0.001
No calcification	252(81.29%)	158(95.76%)	94(64.83%)	
Microcalcification	58(18.71%)	7(4.24%)	51(35.17%)	
***Echostructure***				<0.001
Cystic or mixed	129(41.61%)	99(60.00%)	30 (20.69%)	
solid	181(58.39%)	66(40.00%)	115(79.31%)	

### Logistic Regression Analysis of risk factors in subcentimeter nodules

**Figure 1:**
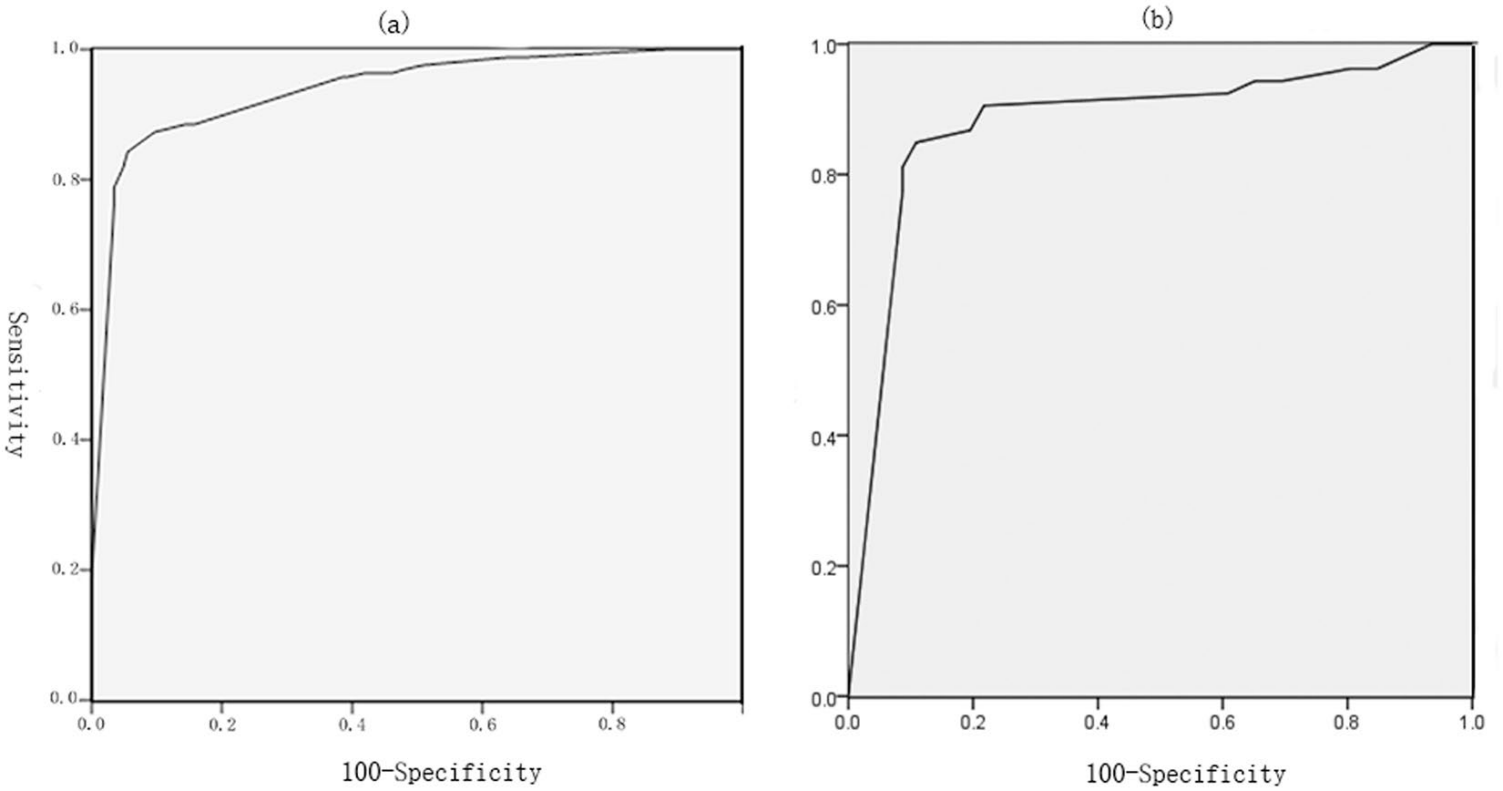
Receiver operating characteristic curve analysis for PTMC. Area under the curve (AUC) estimation for the combined predictive formula in female patients who have given birth to children in training and validation cohorts.

Among the aforementioned risk factors, logistic regression analysis showed that having not more than one boy, taller-than-wide shape, poorly defined margin, marked hypoechogenicity, and microcalcification were independent risk factors for PTMC (training cohort) in multivariate analyses (Table 4). A logistic regression formula was created with the above predictive values. The formula was Logit (P) = 5.672-3.005 (if have not more than one boy)–2.358 (if taller-than-wide shape present) −1.184 (if poorly defined margin present) –2.861 (if marked hypoechogenicity present) −1.860 (if microcalcification present). Based on this formula, a ROC curve was drawn (Fig. 1a). The formula was objective and showed a high predictive value, with an AUC of 0.938. At the predicted probability cutoff value of 0.558, the diagnostic accuracy of the combined predictive formula was 89.03%, which was higher than any other risk factors and conventional US predictive formula (Table 5). The conventional US predictive formula was constructed by the US risk factors including taller-than-wide shape, poorly defined margin, marked hypoechogenicity, and microcalcification. The formula was Logit (P) = 5.603 − 2.346* (if taller-than-wide shape present) −1.222 (if poorly defined margin present) −2.833 (if marked hypoechogenicity present) −1.996 (if microcalcification present). Based on the combined predictive formula, 26 benign nodules (5 thyroiditis and 21 nodular goiter) were misclassified as malignant and 8 PTMCs were misclassified as benign by combined predictive formula. Although the prognostic accuracy of risk factors of fertility condition were lower than those suspicious UC features, the risk factor of having not more than one boy showed the highest sensitivity (97.93%).

**Table 4:** Multivariate binary logistic regression analysis in the prediction of PTMC.

Risk Factors	β	OR (95%CI)	P value
Have not more than one boy	−3.087	0.046(0.005–0.432)	0.007
Taller-than-wide	−2.441	0.087(0.032–0.235)	<0.001
Poorly defined margin	−1.194	0.303(0.116–0.788)	0.014
Marked hypoechogenicity	−3.137	0.043(0.015–0.130)	<0.001
Microcalcification	−1.988	0.137(0.045–0.420)	0.007
Solid			0.648
Age(≥45)			0.060
Diabetes mellitus			0.169
Hypertension			0.071
TSH			0.840
Have at least two girls	—		0.063

CI = confidence interval; TSH = thyroid stimulating hormone.

**Table 5:** Diagnostic performance of conventional US and fertility condition.

Features	Sensitivity (%, 95% CI)	Specificity (%, 95% CI)	PPV (%)	NPV (%)	Accuracy(%)
*Conventional US*					
Taller-than-wide	81.38(73.88–87.17)	84.24(77.58–89.27)	81.94	83.73	82.90
Poorly-defined margins	77.93(70.14–84.21)	84.24(77.58–89.27)	81.29	81.29	81.29
Marked-hypoecogenicity	49.66(41.30–58.03)	95.15(90.34–97.73)	41.62	68.26	73.87
Microcalcification	35.17(27.56–43.59)	95.76(91.11–98.13)	87.93	62.70	67.42
Solid	79.31(71.63–85.40)	60.00(52.07–67.45)	63.54	76.74	69.03
Combined US predictive formula	77.71(70.24–83.79)	84.97(78.09–90.04)	84.14	78.79	81.29
*Sex of children*					
Have not more than one boys	97.93(93.60–99.46)	21.21(15.40–28.40)	55.21	92.11	57.10
Have at least two girls	16.55(11.09–23.83)	96.97(92.70–98.88)	82.76	56.94	59.35
*Combined predictive formula*	94.48(89.06–97.41)	84.24(77.58–89.27)	84.04	94.56	89.03

PPV = positive predictive value; NPV, = negative predictive value.

### Validation of predictive accuracy of the logistic regression model

There was no significant difference in the distribution of baseline characteristics and fertility conditions between the training and validation data sets. The detail information could be found as Supplementary Table S1. The parameters estimated from the training data set were used to predict the probability of being diagnosed with PTMC for the independent validation cohort (80 patients, 99 nodules). Similarly, the predicted probability was used to construct the ROC curve (Fig. 1(b)). The analysis demonstrated that the combined predictive formula had high accuracy in discriminating patients with higher risk of having PTMC from patients with benign nodules (AUC 0.881; 95% CI, 0.807 to 0.956; sensitivity 84.9%, specificity 89.1%).

## Discussion

Although guidelines have declared that active surveillance is the best choice for low-risk PTMC, the diagnostic accuracy is still important. It is reasonable to imagine that patients with malignant nodules could get more attention than benign ones. It is essential to find new ways to increase the diagnostic accuracy of PTMC. This study was designed to investigate the potential predictive factors associated with female PTMC patients who have given birth to a baby. Female patients aged >30 accounted for the majority of patients with thyroid carcinoma^6^, and most of them have babies. Therefore, it is essential to find associated risk factors for these patients, which may be different from other people. We found that fertility condition were significantly associated with PTMC in univariate analyses, including having at least two girls and having not more than one boy. This may be partly elucidated by the difference in leptin levels between women pregnant with female fetuses and women pregnant with male fetuses. Recent report had shown that women with female fetuses have higher maternal serum and cord serum leptin levels compared to women with male fetuses and maternal serum leptin levels are still higher in females who delivered baby girls after delivery^11,12^. In addition, leptin levels were closely related with the development and progression of PTMC^13^. Therefore, it was reasonable to speculate that women who have given birth to two girls may have higher levels of leptin than other people and result in higher incidence of PTMC. Similarly, women who had given birth to fewer boys may have a higher risk of PTMC. It was important to point out that the risk factor of having not more than one boy has the highest prognostic sensitivity compared with US features and having at least two girls. Therefore, it may be simple and effective to ask fertility condition of patients to increase the US diagnosis accuracy of PTMC.

Some reports had shown that patients with elevated thyroid-stimulating hormone (TSH) have higher incidence of thyroid malignancy^14^. In our study, we found that TSH was significantly associated with PTMC in univariate analysis, but it was not independent risk factor in multivariate analyses. This was accord with the result of Prof. Teng W^15^. Similarly, diabetes mellitus and hypertention had been shown to be correlated substantially with PTMC in univariate analyses but not in multivariate analyses.

The combined predictive formula of PTMC had a high accuracy of prognosis. Knowledge of which patients are at higher risk for PTMC may contribute to selection of patients for FNA, institution of risk reduction measures. Based on this study, we suggest ultrasound doctors ask female patients’ fertility condition. Patients who have not more than one boy may have higher risk of PTMC under certain conditions. However, there were some limits for this study. First, this study was not a randomized case-control trial. The conclusions may be limited to the study population. Second, we did not testify the association of differences in leptin levels and fertility condition in our study. It will be investigated in our future study. Third, some unavoidable bias may exist due to the small-scale population. A larger-scale prospective study may be needed to overcome these limitations.

## Patients and Methods

### Patients

The Ethical Committee of Zhejing Provincial People’s hospital, Zhejing, China, approved this retrospective study that was conducted according to the principles of the Declaration of Helsinki. In training cohort, 488 female patients who underwent thyroidectomy from January 1, 2016 to February 1, 2017 in Zhejing Provincial People’s hospital were retrospectively reviewed. In validation cohort, a series of 196 new female patients who underwent thyroidectomy in the same hospital between March 1, 2017 and July 31, 2017 was used to validate the model which was constructed in the training phase. Inclusion criteria were as follows: (1) patients who had a preoperative US diagnosis of thyroid nodules in our hospital and the sonograms could be analyzed by other sonologists; (2) patients who had at least one thyroid nodule smaller than 1 cm in greatest diameter was biopsied; (3) patients who had benign pathologic results and/or papillary thyroid carcinoma. Exclusion criteria were as follows: (1) patients who did not give birth to children. Altogether, a total of 246 patients and 310 nodules (≤1 cm) were included in the training cohort and 80 patients and 99 nodules (≤1 cm) were included in the validation cohort. Cytologic results from FNA were not included for two reasons: (1) part of patients in our hospital refused to undergo preoperative FNA and preferred surgical pathologic examination; and (2) surgical pathologic examination is more effective than FNA for evaluating thyroid nodules of 5 mm or less in diameter. Written information was provided and informed consent was obtained from all subjects.

### US Examination Technique

All patients underwent thyroid US examination within one week before surgery. Examinations were conducted and recorded by a group of skilled sonographers according ACR Thyroid Imaging, Reporting and Data System^16^. Nodules were described according to the following parameters: the maximum diameter; partially cystic echostructure or solid echostructure; none, marked hypo-, hypo-, iso-, or hyper-echogenicity; with or without microcalcification; well-defined or poorly-defined margins; wider-than-tall or taller-than-wide shape. FNA was performed only for patients with suspicious US features.

### Statistical Analysis

We evaluate the potential clinical risk factors and conventional US features associated with PTMC in univariate analysis. Continuous variables were expressed as mean ± standard deviation with evaluated by Student’s t test, and categoric variables were presented as frequency and percentages with analyzed by X2 test or Fisher’s exact test when appropriate. Risk factors were considered significant if the P-value ≤0.10 and they would be incorporated into the logistic regression analysis. Multivariate analyses of risk factors for PTMC were carried out with logistic regression using forward selection algorithm. In these analyses, all probabilities were two-tailed and P values less than 0.05 were regarded as statistically significant. All statistical analyses were performed using the SPSS (version 17.0, SPSS Inc., Chicago, IL).

## Electronic supplementary material

Table S1
