# Author Correction: Diagnostic value of ultrasound features and sex of fetuses in female patients with papillary thyroid microcarcinoma

**DOI:** 10.1038/s41598-018-31340-6

**Published:** 2018-09-03

**Authors:** Chun-jie Hou, Ran Wei, Jing-lan Tang, Qiao-hong Hu, Hong-feng He, Xiao-ming Fan

**Affiliations:** 1 Department of Ultrasound, Zhejiang Provincial People’s Hospital, Hangzhou, China; 2 People’s Hospital of Hangzhou Medical College, Hangzhou, China; 3 Department of General Surgery, Huashan Hospital & Cancer Metastasis Institute, Fudan University, Shanghai, China

Correction to: *Scientific Reports* 10.1038/s41598-018-26003-5, published online 14 May 2018

The original version of this Article contained an error in Affiliation 1, which was incorrectly given as ‘Department of Ultrasound, Zhejing Provincial People’s Hospital, Hangzhou, China’. The correct affiliation is listed below:

Department of Ultrasound, Zhejiang Provincial People’s Hospital, Hangzhou, China

This error has now been corrected in the HTML and PDF versions of the Article, and in the accompanying Supplementary information.

