# Supplementary material for: Diagnostic value of ultrasound features and sex of fetuses in female patients with papillary thyroid microcarcinoma
Source: Sci Rep. 2018 May 14;8:7510. doi: 10.1038/s41598-018-26003-5 (PMC5951819; doi:10.1038/s41598-018-26003-5)
Supplement: Supplementary file 1 — Table S1 [file 41598_2018_26003_MOESM1_ESM.docx]

**Supplementary Information**

**Diagnostic value of ultrasound features and sex of fetuses in female patients with papillary thyroid microcarcinoma**

**Chun-jie Hou^1,2,^*, Ran Wei^3,^*, Jing-lan Tang^1,2,&^, Qiao-hong Hu^1,2^, Hong-feng He^1,2^,** **Xiao-ming Fan^1,2^**

^1^Department of Ultrasound, Zhejiang Provincial People’s Hospital, Hangzhou, China

^2^People’s Hospital of Hangzhou Medical College, Hangzhou, China

^3^Department of General Surgery, Huashan Hospital & Cancer Metastasis Institute, Fudan University, Shanghai, China

*These authors contributed equally to this work

**^&^**Corresponding author: Jing-lan Tang( [tangjinglan＿85@163.com](about:blank))

| Viariables^a^ | Training  (N=246) | Validation  (N=80) | *P* value |
| --- | --- | --- | --- |
| Age(≥45) | 183(74.39%) | 52(65.00%) | 0.115 |
| Body mass index(kg/m^2^) | 24.73±14.33 | 23.91±3.43 | 0.616 |
| Hypertension | 58(23.58%) | 15(18.75%) | 0.441 |
| Diabetes mellitus | 28(11.38%) | 7(8.75%) | 0.544 |
| TSH | 1.80±1.25 | 2.03±1.52 | 0.187 |
| Glucose | 5.38±1.08 | 5.48±1.08 | 0.146 |
| TC | 5.03±0.94 | 5.05±1.05 | 0.889 |
| TG | 1.53±1.24 | 1.86±2.24 | 0.234 |
| HDL | 1.35±0.31 | 1.32±0.34 | 0.406 |
| LDL | 2.95±0.73 | 2.92±0.69 | 0.736 |
| LDH | 174.49±33.79 | 176.39±34.80 | 0.671 |
| Have at least two girls | 40(16.26%) | 16(20.00%) | 0.495 |
| Have not more than one boy | 29(11.79%) | 11(13.75%) | 0.695 |

**Supplementary Table S1. Characteristics of study participants in the training and validation data sets.**  ^a^ Continuous data are shown as mean ± standard deviation; categoric data as number (%).
